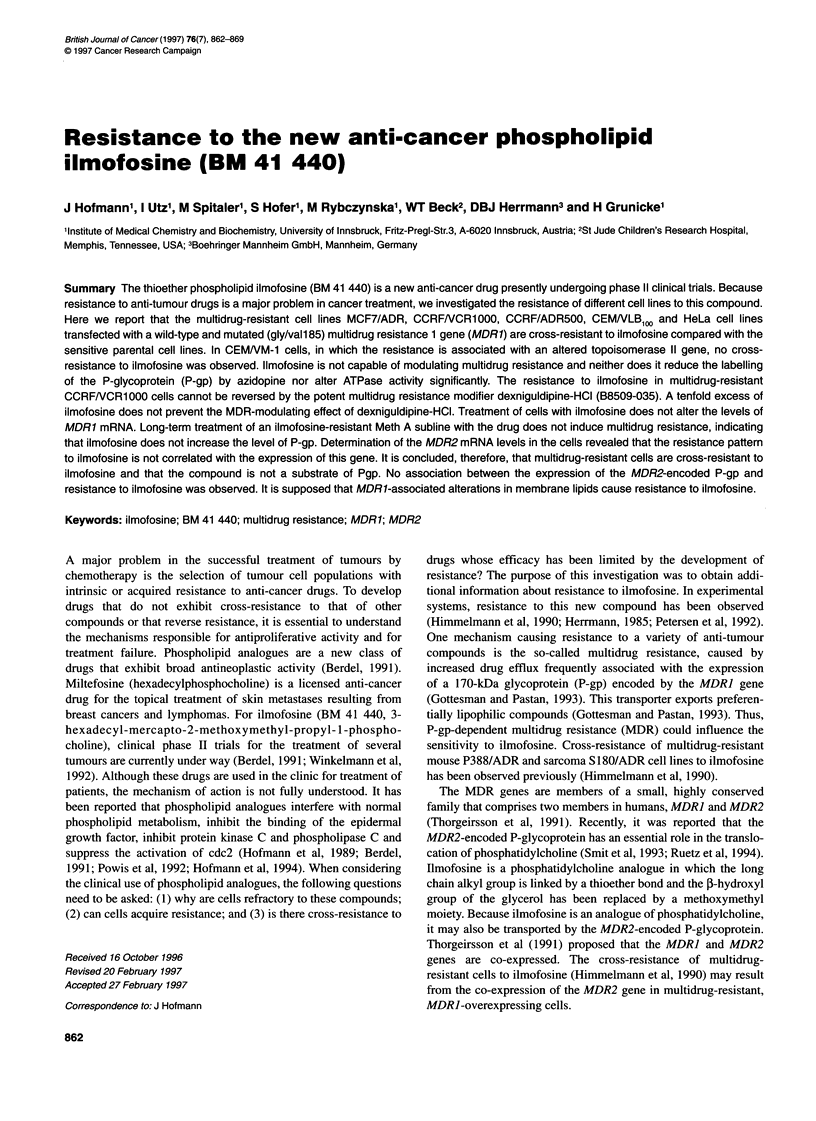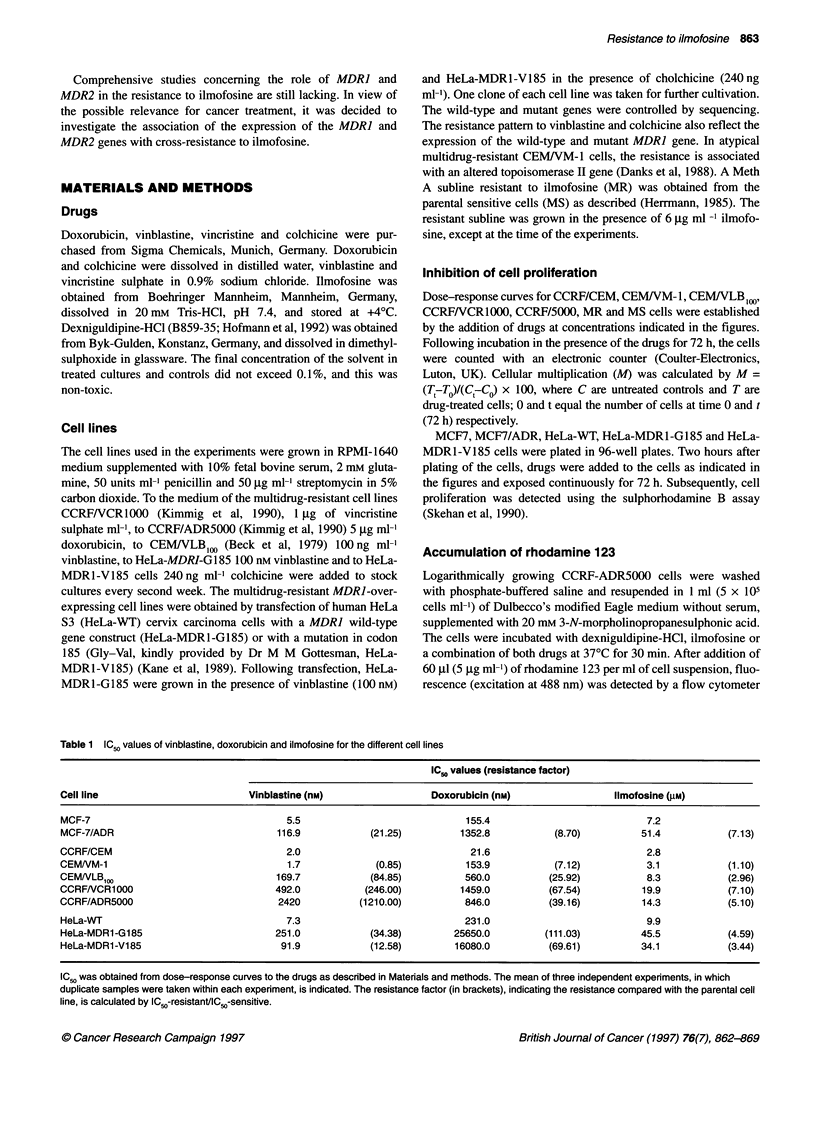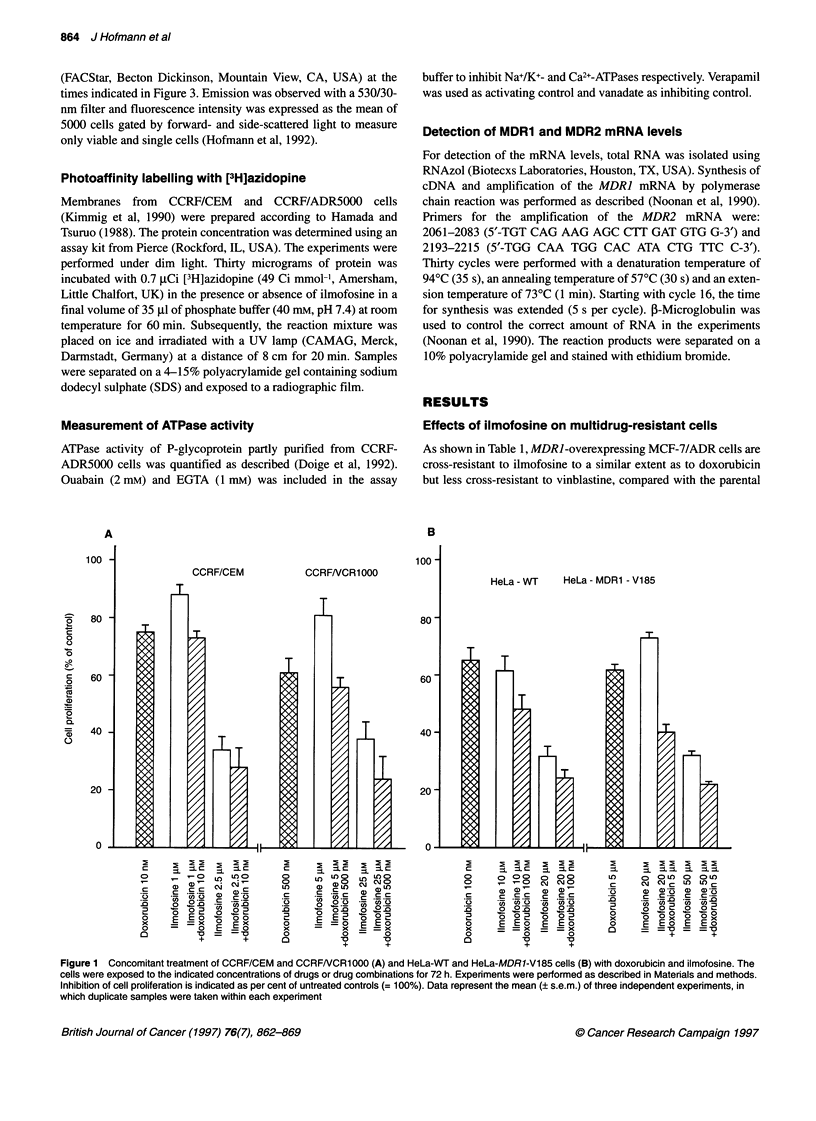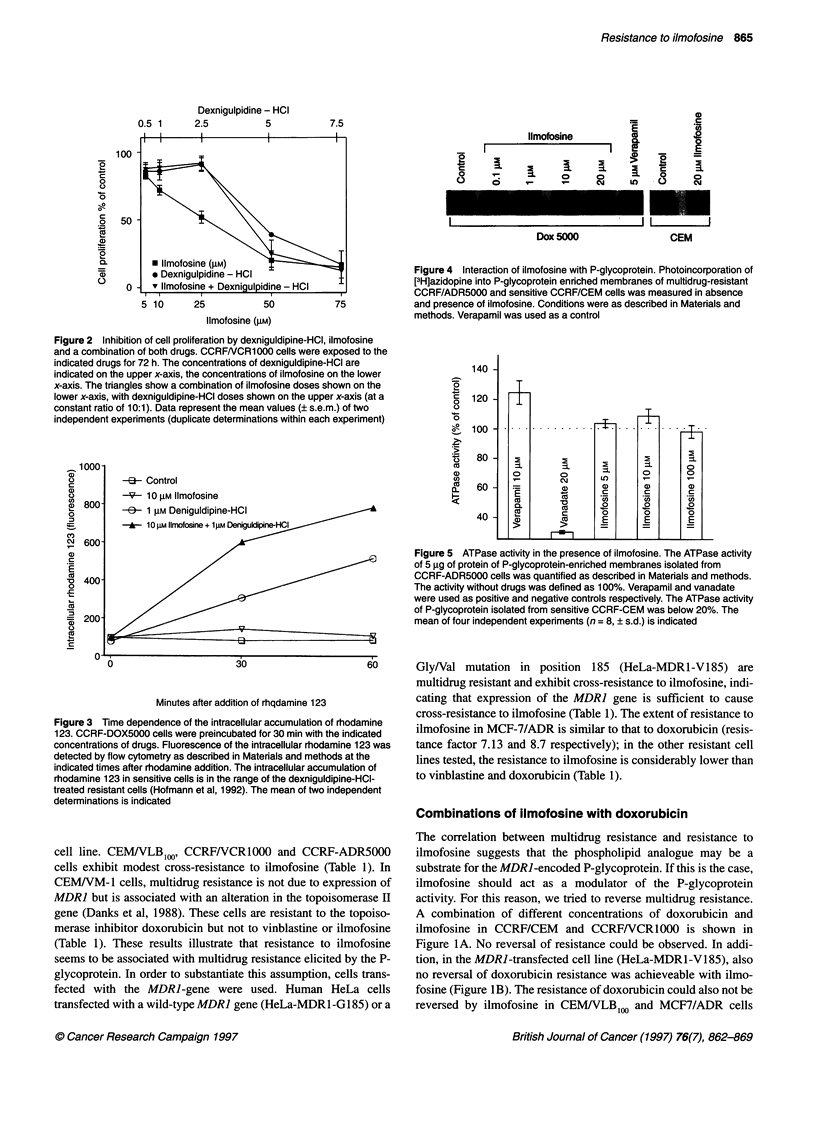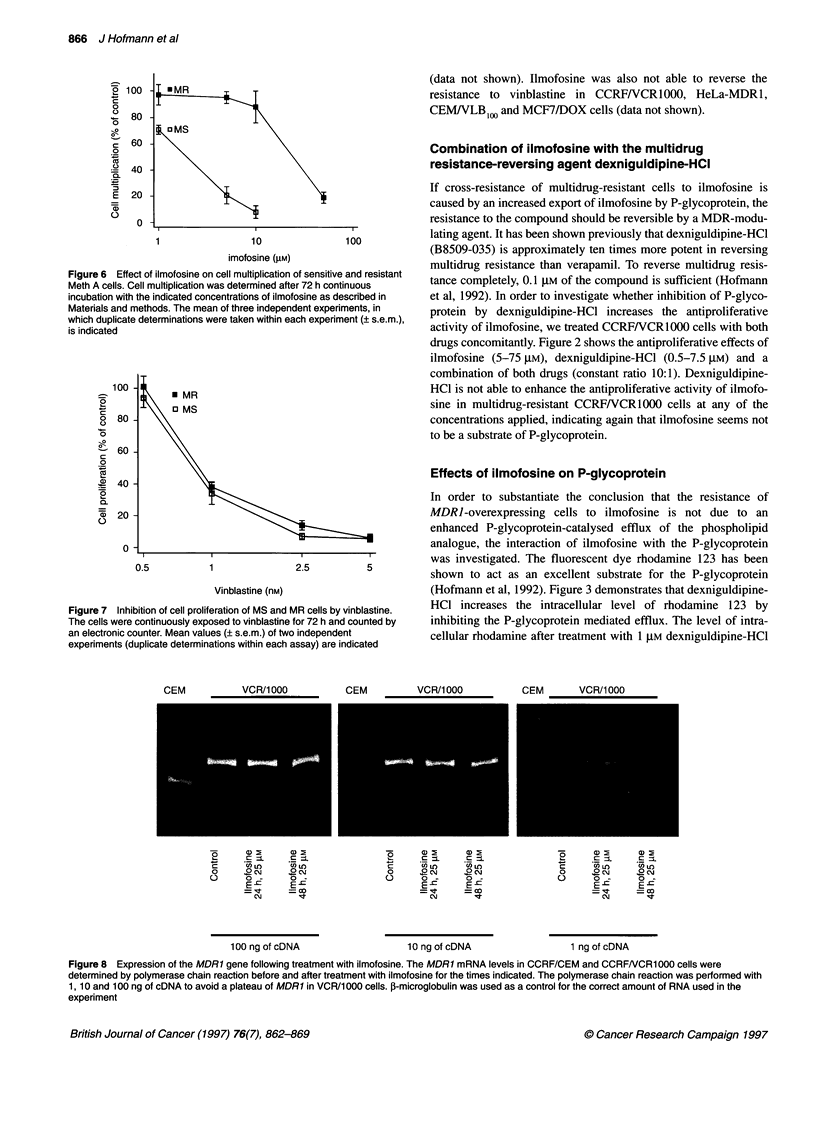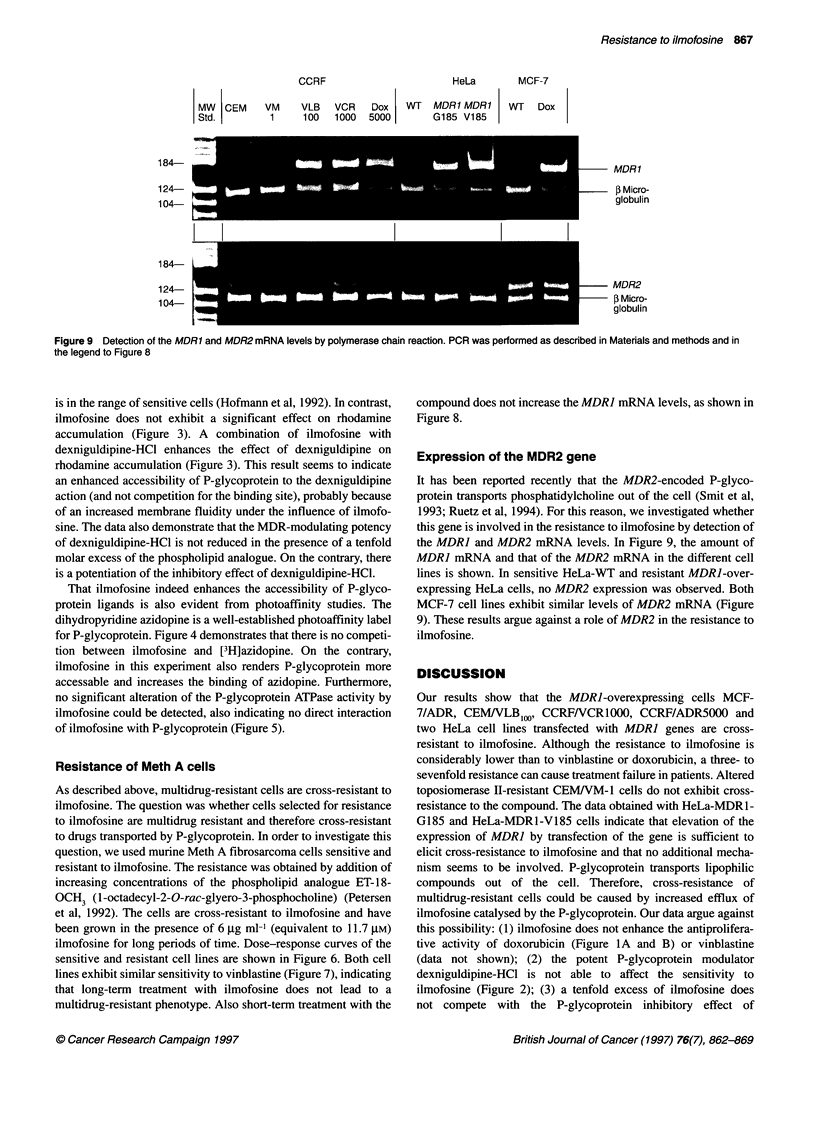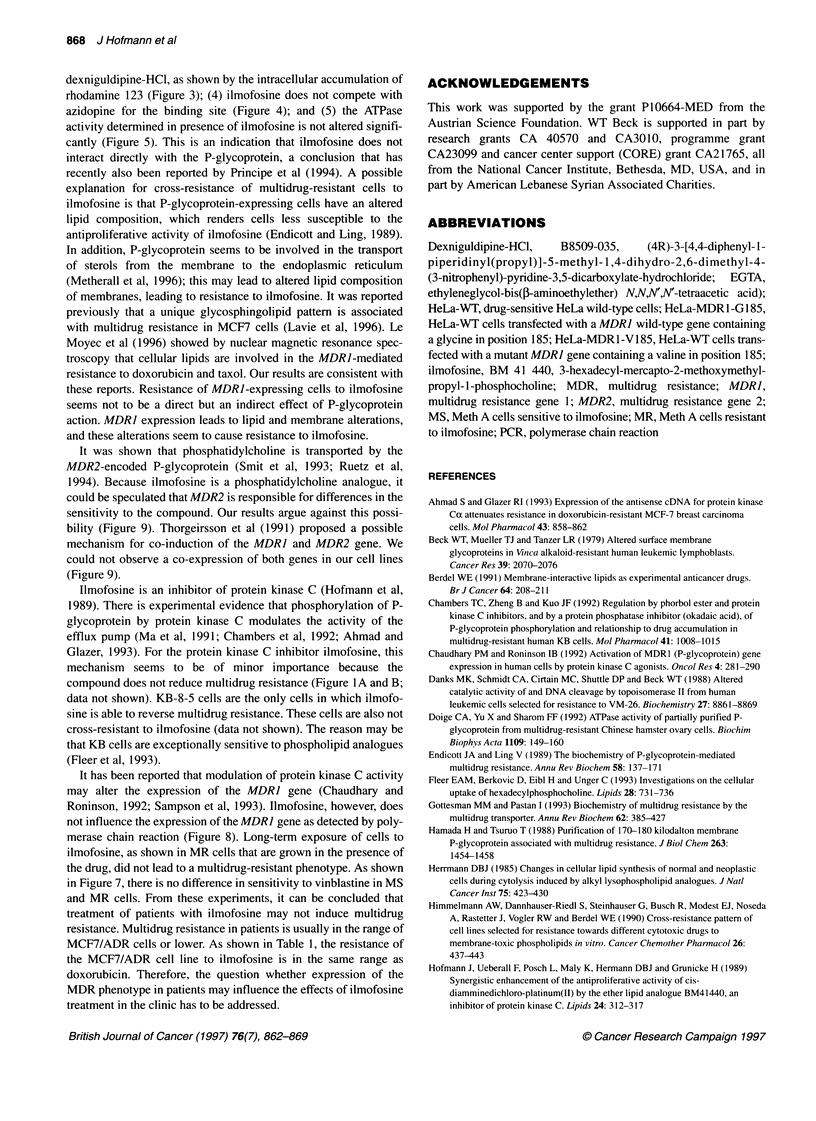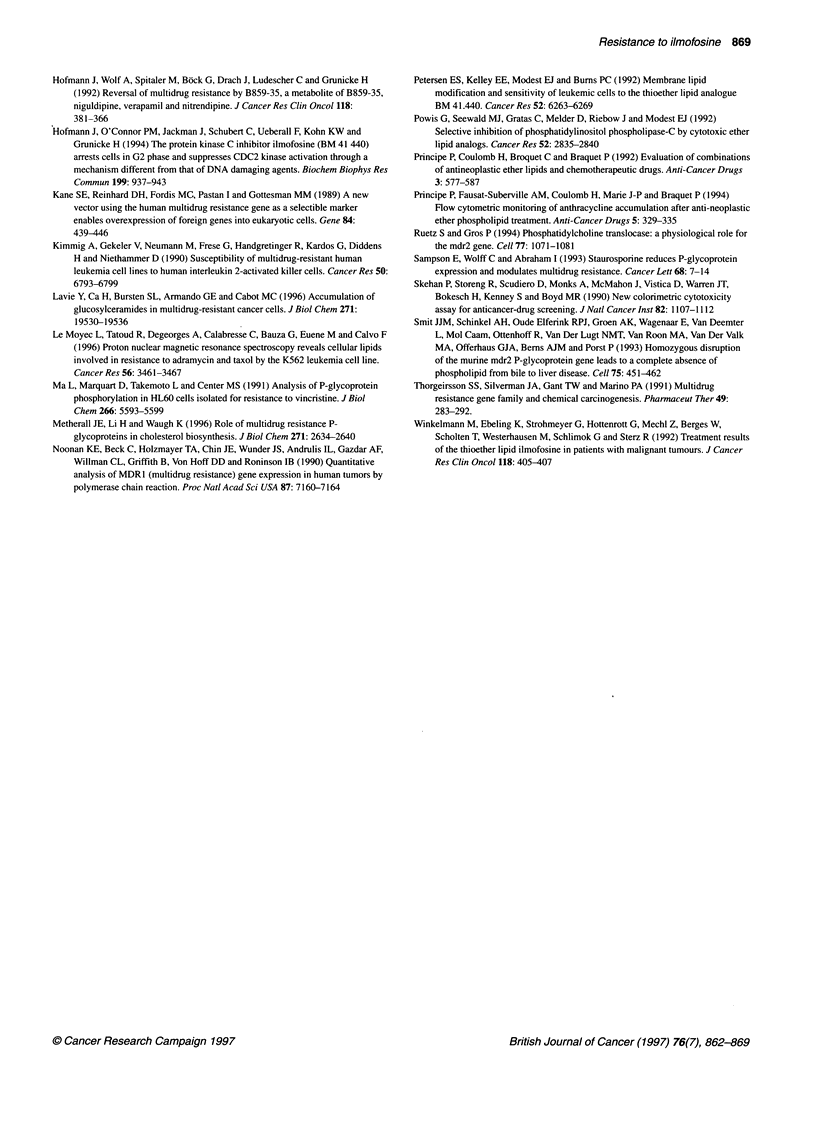# Resistance to the new anti-cancer phospholipid ilmofosine (BM 41 440).

**DOI:** 10.1038/bjc.1997.476

**Published:** 1997

**Authors:** J. Hofmann, I. Utz, M. Spitaler, S. Hofer, M. Rybczynska, W. T. Beck, D. B. Herrmann, H. Grunicke

**Affiliations:** Institute of Medical Chemistry and Biochemistry, University of Innsbruck, Austria.

## Abstract

**Images:**


British Journal of Cancer (1997) 76(7), 862–869
© 1997 Cancer Research Campaign

### Resistance to the new anti-cancer phospholipid ilmofosine (BM 41 440)

**J Hofmann[1], I Utz[1], M Spitaler[1], S Hofer[1], M Rybczynska[1], WT Beck[2], DBJ Herrmann[3] and H Grunicke[1]**

[1]Institute of Medical Chemistry and Biochemistry, University of Innsbruck, Fritz-Pregl-Str.3, A-6020 Innsbruck, Austria; [2]St Jude Children's Research Hospital, Memphis, Tennessee, USA; [3]Boehringer Mannheim GmbH, Mannheim, Germany

**Summary** The thioether phospholipid ilmofosine (BM 41 440) is a new anti-cancer drug presently undergoing phase II clinical trials. Because resistance to anti-tumour drugs is a major problem in cancer treatment, we investigated the resistance of different cell lines to this compound. Here we report that the multidrug-resistant cell lines MCF7/ADR, CCRF/VCR1000, CCRF/ADR500, CEM/$VLB_{100}$ and HeLa cell lines transfected with a wild-type and mutated (gly/val185) multidrug resistance 1 gene (*MDR1*) are cross-resistant to ilmofosine compared with the sensitive parental cell lines. In CEM/VM-1 cells, in which the resistance is associated with an altered topoisomerase II gene, no cross-resistance to ilmofosine was observed. Ilmofosine is not capable of modulating multidrug resistance and neither does it reduce the labelling of the P-glycoprotein (P-gp) by azidopine nor alter ATPase activity significantly. The resistance to ilmofosine in multidrug-resistant CCRF/VCR1000 cells cannot be reversed by the potent multidrug resistance modifier dexniguldipine-HCl (B8509-035). A tenfold excess of ilmofosine does not prevent the MDR-modulating effect of dexniguldipine-HCl. Treatment of cells with ilmofosine does not alter the levels of *MDR1* mRNA. Long-term treatment of an ilmofosine-resistant Meth A subline with the drug does not induce multidrug resistance, indicating that ilmofosine does not increase the level of P-gp. Determination of the *MDR2* mRNA levels in the cells revealed that the resistance pattern to ilmofosine is not correlated with the expression of this gene. It is concluded, therefore, that multidrug-resistant cells are cross-resistant to ilmofosine and that the compound is not a substrate of Pgp. No association between the expression of the *MDR2*-encoded P-gp and resistance to ilmofosine was observed. It is supposed that *MDR1*-associated alterations in membrane lipids cause resistance to ilmofosine.

**Keywords:** ilmofosine; BM 41 440; multidrug resistance; *MDR1*; *MDR2*

A major problem in the successful treatment of tumours by chemotherapy is the selection of tumour cell populations with intrinsic or acquired resistance to anti-cancer drugs. To develop drugs that do not exhibit cross-resistance to that of other compounds or that reverse resistance, it is essential to understand the mechanisms responsible for antiproliferative activity and for treatment failure. Phospholipid analogues are a new class of drugs that exhibit broad antineoplastic activity (Berdel, 1991). Miltefosine (hexadecylphosphocholine) is a licensed anti-cancer drug for the topical treatment of skin metastases resulting from breast cancers and lymphomas. For ilmofosine (BM 41 440, 3-hexadecyl-mercapto-2-methoxymethyl-propyl-1-phosphocholine), clinical phase II trials for the treatment of several tumours are currently under way (Berdel, 1991; Winkelmann et al, 1992). Although these drugs are used in the clinic for treatment of patients, the mechanism of action is not fully understood. It has been reported that phospholipid analogues interfere with normal phospholipid metabolism, inhibit the binding of the epidermal growth factor, inhibit protein kinase C and phospholipase C and suppress the activation of cdc2 (Hofmann et al, 1989; Berdel, 1991; Powis et al, 1992; Hofmann et al, 1994). When considering the clinical use of phospholipid analogues, the following questions need to be asked: (1) why are cells refractory to these compounds; (2) can cells acquire resistance; and (3) is there cross-resistance to drugs whose efficacy has been limited by the development of resistance? The purpose of this investigation was to obtain additional information about resistance to ilmofosine. In experimental systems, resistance to this new compound has been observed (Himmelmann et al, 1990; Herrmann, 1985; Petersen et al, 1992). One mechanism causing resistance to a variety of anti-tumour compounds is the so-called multidrug resistance, caused by increased drug efflux frequently associated with the expression of a 170-kDa glycoprotein (P-gp) encoded by the *MDR1* gene (Gottesman and Pastan, 1993). This transporter exports preferentially lipophilic compounds (Gottesman and Pastan, 1993). Thus, P-gp-dependent multidrug resistance (MDR) could influence the sensitivity to ilmofosine. Cross-resistance of multidrug-resistant mouse P388/ADR and sarcoma S180/ADR cell lines to ilmofosine has been observed previously (Himmelmann et al, 1990).

The MDR genes are members of a small, highly conserved family that comprises two members in humans, *MDR1* and *MDR2* (Thorgeirsson et al, 1991). Recently, it was reported that the *MDR2*-encoded P-glycoprotein has an essential role in the translocation of phosphatidylcholine (Smit et al, 1993; Ruetz et al, 1994). Ilmofosine is a phosphatidylcholine analogue in which the long chain alkyl group is linked by a thioether bond and the β-hydroxyl group of the glycerol has been replaced by a methoxymethyl moiety. Because ilmofosine is an analogue of phosphatidylcholine, it may also be transported by the *MDR2*-encoded P-glycoprotein. Thorgeirsson et al (1991) proposed that the *MDR1* and *MDR2* genes are co-expressed. The cross-resistance of multidrug-resistant cells to ilmofosine (Himmelmann et al, 1990) may result from the co-expression of the *MDR2* gene in multidrug-resistant, *MDR1*-overexpressing cells.

*Received 16 October 1996*
*Revised 20 February 1997*
*Accepted 27 February 1997*

*Correspondence to:* J Hofmann

Comprehensive studies concerning the role of *MDR1* and *MDR2* in the resistance to ilmofosine are still lacking. In view of the possible relevance for cancer treatment, it was decided to investigate the association of the expression of the *MDR1* and *MDR2* genes with cross-resistance to ilmofosine.

### MATERIALS AND METHODS

### Drugs

Doxorubicin, vinblastine, vincristine and colchicine were purchased from Sigma Chemicals, Munich, Germany. Doxorubicin and colchicine were dissolved in distilled water, vinblastine and vincristine sulphate in 0.9% sodium chloride. Ilmofosine was obtained from Boehringer Mannheim, Mannheim, Germany, dissolved in 20 mM Tris-HCl, pH 7.4, and stored at +4°C. Dexniguldipine-HCl (B859-35; Hofmann et al, 1992) was obtained from Byk-Gulden, Konstanz, Germany, and dissolved in dimethylsulphoxide in glassware. The final concentration of the solvent in treated cultures and controls did not exceed 0.1%, and this was non-toxic.

### Cell lines

The cell lines used in the experiments were grown in RPMI-1640 medium supplemented with 10% fetal bovine serum, 2 mM glutamine, 50 units $ml^{-1}$ penicillin and 50 µg $ml^{-1}$ streptomycin in 5% carbon dioxide. To the medium of the multidrug-resistant cell lines CCRF/VCR1000 (Kimmig et al, 1990), 1 µg of vincristine sulphate $ml^{-1}$, to CCRF/ADR5000 (Kimmig et al, 1990) 5 µg $ml^{-1}$ doxorubicin, to CEM/$VLB_{100}$ (Beck et al, 1979) 100 ng $ml^{-1}$ vinblastine, to HeLa-*MDR1*-G185 100 nM vinblastine and to HeLa-MDR1-V185 cells 240 ng $ml^{-1}$ colchicine were added to stock cultures every second week. The multidrug-resistant *MDR1*-overexpressing cell lines were obtained by transfection of human HeLa S3 (HeLa-WT) cervix carcinoma cells with a *MDR1* wild-type gene construct (HeLa-MDR1-G185) or with a mutation in codon 185 (Gly–Val, kindly provided by Dr M M Gottesman, HeLa-MDR1-V185) (Kane et al, 1989). Following transfection, HeLa-MDR1-G185 were grown in the presence of vinblastine (100 nM) and HeLa-MDR1-V185 in the presence of cholchicine (240 ng $ml^{-1}$). One clone of each cell line was taken for further cultivation. The wild-type and mutant genes were controlled by sequencing. The resistance pattern to vinblastine and colchicine also reflect the expression of the wild-type and mutant *MDR1* gene. In atypical multidrug-resistant CEM/VM-1 cells, the resistance is associated with an altered topoisomerase II gene (Danks et al, 1988). A Meth A subline resistant to ilmofosine (MR) was obtained from the parental sensitive cells (MS) as described (Herrmann, 1985). The resistant subline was grown in the presence of 6 µg $ml^{-1}$ ilmofosine, except at the time of the experiments.

### Inhibition of cell proliferation

Dose–response curves for CCRF/CEM, CEM/VM-1, CEM/$VLB_{100}$, CCRF/VCR1000, CCRF/5000, MR and MS cells were established by the addition of drugs at concentrations indicated in the figures. Following incubation in the presence of the drugs for 72 h, the cells were counted with an electronic counter (Coulter-Electronics, Luton, UK). Cellular multiplication (*M*) was calculated by $M = (T_t - T_0)/(C_t - C_0) \times 100$, where *C* are untreated controls and *T* are drug-treated cells; 0 and t equal the number of cells at time 0 and *t* (72 h) respectively.

MCF7, MCF7/ADR, HeLa-WT, HeLa-MDR1-G185 and HeLa-MDR1-V185 cells were plated in 96-well plates. Two hours after plating of the cells, drugs were added to the cells as indicated in the figures and exposed continuously for 72 h. Subsequently, cell proliferation was detected using the sulphorhodamine B assay (Skehan et al, 1990).

### Accumulation of rhodamine 123

Logarithmically growing CCRF-ADR5000 cells were washed with phosphate-buffered saline and resupended in 1 ml ($5 \times 10^5$ cells $ml^{-1}$) of Dulbecco's modified Eagle medium without serum, supplemented with 20 mM 3-*N*-morpholinopropanesulphonic acid. The cells were incubated with dexniguldipine-HCl, ilmofosine or a combination of both drugs at 37°C for 30 min. After addition of 60 µl (5 µg $ml^{-1}$) of rhodamine 123 per ml of cell suspension, fluorescence (excitation at 488 nm) was detected by a flow cytometer

**Table 1** $IC_{50}$ values of vinblastine, doxorubicin and ilmofosine for the different cell lines

| | $IC_{50}$ values (resistance factor) | | | | | |
|---|---|---|---|---|---|---|
| **Cell line** | **Vinblastine (nM)** | | **Doxorubicin (nM)** | | **Ilmofosine (µM)** | |
| MCF-7 | 5.5 | | 155.4 | | 7.2 | |
| MCF-7/ADR | 116.9 | (21.25) | 1352.8 | (8.70) | 51.4 | (7.13) |
| CCRF/CEM | 2.0 | | 21.6 | | 2.8 | |
| CEM/VM-1 | 1.7 | (0.85) | 153.9 | (7.12) | 3.1 | (1.10) |
| CEM/$VLB_{100}$ | 169.7 | (84.85) | 560.0 | (25.92) | 8.3 | (2.96) |
| CCRF/VCR1000 | 492.0 | (246.00) | 1459.0 | (67.54) | 19.9 | (7.10) |
| CCRF/ADR5000 | 2420 | (1210.00) | 846.0 | (39.16) | 14.3 | (5.10) |
| HeLa-WT | 7.3 | | 231.0 | | 9.9 | |
| HeLa-MDR1-G185 | 251.0 | (34.38) | 25650.0 | (111.03) | 45.5 | (4.59) |
| HeLa-MDR1-V185 | 91.9 | (12.58) | 16080.0 | (69.61) | 34.1 | (3.44) |

$IC_{50}$ was obtained from dose–response curves to the drugs as described in Materials and methods. The mean of three independent experiments, in which duplicate samples were taken within each experiment, is indicated. The resistance factor (in brackets), indicating the resistance compared with the parental cell line, is calculated by $IC_{50}$-resistant/$IC_{50}$-sensitive.

© Cancer Research Campaign 1997

(FACStar, Becton Dickinson, Mountain View, CA, USA) at the times indicated in Figure 3. Emission was observed with a 530/30-nm filter and fluorescence intensity was expressed as the mean of 5000 cells gated by forward- and side-scattered light to measure only viable and single cells (Hofmann et al, 1992).

### Photoaffinity labelling with [$^3$H]azidopine

Membranes from CCRF/CEM and CCRF/ADR5000 cells (Kimmig et al, 1990) were prepared according to Hamada and Tsuruo (1988). The protein concentration was determined using an assay kit from Pierce (Rockford, IL, USA). The experiments were performed under dim light. Thirty micrograms of protein was incubated with 0.7 μCi [$^3$H]azidopine (49 Ci mmol$^{-1}$, Amersham, Little Chalfort, UK) in the presence or absence of ilmofosine in a final volume of 35 μl of phosphate buffer (40 mM, pH 7.4) at room temperature for 60 min. Subsequently, the reaction mixture was placed on ice and irradiated with a UV lamp (CAMAG, Merck, Darmstadt, Germany) at a distance of 8 cm for 20 min. Samples were separated on a 4–15% polyacrylamide gel containing sodium dodecyl sulphate (SDS) and exposed to a radiographic film.

### Measurement of ATPase activity

ATPase activity of P-glycoprotein partly purified from CCRF-ADR5000 cells was quantified as described (Doige et al, 1992). Ouabain (2 mM) and EGTA (1 mM) was included in the assay buffer to inhibit $Na^+/K^+$- and $Ca^{2+}$-ATPases respectively. Verapamil was used as activating control and vanadate as inhibiting control.

### Detection of MDR1 and MDR2 mRNA levels

For detection of the mRNA levels, total RNA was isolated using RNAzol (Biotecxs Laboratories, Houston, TX, USA). Synthesis of cDNA and amplification of the *MDR1* mRNA by polymerase chain reaction was performed as described (Noonan et al, 1990). Primers for the amplification of the *MDR2* mRNA were: 2061–2083 (5′-TGT CAG AAG AGC CTT GAT GTG G-3′) and 2193–2215 (5′-TGG CAA TGG CAC ATA CTG TTC C-3′). Thirty cycles were performed with a denaturation temperature of 94°C (35 s), an annealing temperature of 57°C (30 s) and an extension temperature of 73°C (1 min). Starting with cycle 16, the time for synthesis was extended (5 s per cycle). β-Microglobulin was used to control the correct amount of RNA in the experiments (Noonan et al, 1990). The reaction products were separated on a 10% polyacrylamide gel and stained with ethidium bromide.

### RESULTS

### Effects of ilmofosine on multidrug-resistant cells

As shown in Table 1, *MDR1*-overexpressing MCF-7/ADR cells are cross-resistant to ilmofosine to a similar extent as to doxorubicin but less cross-resistant to vinblastine, compared with the parental

**Figure 1** Concomitant treatment of CCRF/CEM and CCRF/VCR1000 (**A**) and HeLa-WT and HeLa-*MDR1*-V185 cells (**B**) with doxorubicin and ilmofosine. The cells were exposed to the indicated concentrations of drugs or drug combinations for 72 h. Experiments were performed as described in Materials and methods. Inhibition of cell proliferation is indicated as per cent of untreated controls (= 100%). Data represent the mean (± s.e.m.) of three independent experiments, in which duplicate samples were taken within each experiment

 © Cancer Research Campaign 1997

**Figure 2** Inhibition of cell proliferation by dexniguldipine-HCl, ilmofosine and a combination of both drugs. CCRF/VCR1000 cells were exposed to the indicated drugs for 72 h. The concentrations of dexniguldipine-HCl are indicated on the upper *x*-axis, the concentrations of ilmofosine on the lower *x*-axis. The triangles show a combination of ilmofosine doses shown on the lower *x*-axis, with dexniguldipine-HCl doses shown on the upper *x*-axis (at a constant ratio of 10:1). Data represent the mean values (± s.e.m.) of two independent experiments (duplicate determinations within each experiment)

**Figure 3** Time dependence of the intracellular accumulation of rhodamine 123. CCRF-DOX5000 cells were preincubated for 30 min with the indicated concentrations of drugs. Fluorescence of the intracellular rhodamine 123 was detected by flow cytometry as described in Materials and methods at the indicated times after rhodamine addition. The intracellular accumulation of rhodamine 123 in sensitive cells is in the range of the dexniguldipine-HCl-treated resistant cells (Hofmann et al, 1992). The mean of two independent determinations is indicated

**Figure 4** Interaction of ilmofosine with P-glycoprotein. Photoincorporation of [$^3$H]azidopine into P-glycoprotein enriched membranes of multidrug-resistant CCRF/ADR5000 and sensitive CCRF/CEM cells was measured in absence and presence of ilmofosine. Conditions were as described in Materials and methods. Verapamil was used as a control

**Figure 5** ATPase activity in the presence of ilmofosine. The ATPase activity of 5 μg of protein of P-glycoprotein-enriched membranes isolated from CCRF-ADR5000 cells was quantified as described in Materials and methods. The activity without drugs was defined as 100%. Verapamil and vanadate were used as positive and negative controls respectively. The ATPase activity of P-glycoprotein isolated from sensitive CCRF-CEM was below 20%. The mean of four independent experiments ($n$ = 8, ± s.d.) is indicated cell line. CEM/VLB$_{100}$, CCRF/VCR1000 and CCRF-ADR5000 cells exhibit modest cross-resistance to ilmofosine (Table 1). In CEM/VM-1 cells, multidrug resistance is not due to expression of *MDR1* but is associated with an alteration in the topoisomerase II gene (Danks et al, 1988). These cells are resistant to the topoisomerase inhibitor doxorubicin but not to vinblastine or ilmofosine (Table 1). These results illustrate that resistance to ilmofosine seems to be associated with multidrug resistance elicited by the P-glycoprotein. In order to substantiate this assumption, cells transfected with the *MDR1*-gene were used. Human HeLa cells transfected with a wild-type *MDR1* gene (HeLa-MDR1-G185) or a Gly/Val mutation in position 185 (HeLa-MDR1-V185) are multidrug resistant and exhibit cross-resistance to ilmofosine, indicating that expression of the *MDR1* gene is sufficient to cause cross-resistance to ilmofosine (Table 1). The extent of resistance to ilmofosine in MCF-7/ADR is similar to that to doxorubicin (resistance factor 7.13 and 8.7 respectively); in the other resistant cell lines tested, the resistance to ilmofosine is considerably lower than to vinblastine and doxorubicin (Table 1).

### Combinations of ilmofosine with doxorubicin

The correlation between multidrug resistance and resistance to ilmofosine suggests that the phospholipid analogue may be a substrate for the *MDR1*-encoded P-glycoprotein. If this is the case, ilmofosine should act as a modulator of the P-glycoprotein activity. For this reason, we tried to reverse multidrug resistance. A combination of different concentrations of doxorubicin and ilmofosine in CCRF/CEM and CCRF/VCR1000 is shown in Figure 1A. No reversal of resistance could be observed. In addition, in the *MDR1*-transfected cell line (HeLa-MDR1-V185), also no reversal of doxorubicin resistance was achieveable with ilmofosine (Figure 1B). The resistance of doxorubicin could also not be reversed by ilmofosine in CEM/VLB$_{100}$ and MCF7/ADR cells

© Cancer Research Campaign 1997

**Figure 6** Effect of ilmofosine on cell multiplication of sensitive and resistant Meth A cells. Cell multiplication was determined after 72 h continuous incubation with the indicated concentrations of ilmofosine as described in Materials and methods. The mean of three independent experiments, in which duplicate determinations were taken within each experiment (± s.e.m.), is indicated

**Figure 7** Inhibition of cell proliferation of MS and MR cells by vinblastine. The cells were continuously exposed to vinblastine for 72 h and counted by an electronic counter. Mean values (± s.e.m.) of two independent experiments (duplicate determinations within each assay) are indicated

(data not shown). Ilmofosine was also not able to reverse the resistance to vinblastine in CCRF/VCR1000, HeLa-MDR1, CEM/VLB$_{100}$ and MCF7/DOX cells (data not shown).

### Combination of ilmofosine with the multidrug resistance-reversing agent dexniguldipine-HCl

If cross-resistance of multidrug-resistant cells to ilmofosine is caused by an increased export of ilmofosine by P-glycoprotein, the resistance to the compound should be reversible by a MDR-modulating agent. It has been shown previously that dexniguldipine-HCl (B8509-035) is approximately ten times more potent in reversing multidrug resistance than verapamil. To reverse multidrug resistance completely, 0.1 μM of the compound is sufficient (Hofmann et al, 1992). In order to investigate whether inhibition of P-glycoprotein by dexniguldipine-HCl increases the antiproliferative activity of ilmofosine, we treated CCRF/VCR1000 cells with both drugs concomitantly. Figure 2 shows the antiproliferative effects of ilmofosine (5–75 μM), dexniguldipine-HCl (0.5–7.5 μM) and a combination of both drugs (constant ratio 10:1). Dexniguldipine-HCl is not able to enhance the antiproliferative activity of ilmofosine in multidrug-resistant CCRF/VCR1000 cells at any of the concentrations applied, indicating again that ilmofosine seems not to be a substrate of P-glycoprotein.

### Effects of ilmofosine on P-glycoprotein

In order to substantiate the conclusion that the resistance of *MDR1*-overexpressing cells to ilmofosine is not due to an enhanced P-glycoprotein-catalysed efflux of the phospholipid analogue, the interaction of ilmofosine with the P-glycoprotein was investigated. The fluorescent dye rhodamine 123 has been shown to act as an excellent substrate for the P-glycoprotein (Hofmann et al, 1992). Figure 3 demonstrates that dexniguldipine-HCl increases the intracellular level of rhodamine 123 by inhibiting the P-glycoprotein mediated efflux. The level of intracellular rhodamine after treatment with 1 μM dexniguldipine-HCl

**Figure 8** Expression of the *MDR1* gene following treatment with ilmofosine. The *MDR1* mRNA levels in CCRF/CEM and CCRF/VCR1000 cells were determined by polymerase chain reaction before and after treatment with ilmofosine for the times indicated. The polymerase chain reaction was performed with 1, 10 and 100 ng of cDNA to avoid a plateau of *MDR1* in VCR/1000 cells. β-microglobulin was used as a control for the correct amount of RNA used in the experiment

© Cancer Research Campaign 1997

**Figure 9** Detection of the *MDR1* and *MDR2* mRNA levels by polymerase chain reaction. PCR was performed as described in Materials and methods and in the legend to Figure 8

is in the range of sensitive cells (Hofmann et al, 1992). In contrast, ilmofosine does not exhibit a significant effect on rhodamine accumulation (Figure 3). A combination of ilmofosine with dexniguldipine-HCl enhances the effect of dexniguldipine on rhodamine accumulation (Figure 3). This result seems to indicate an enhanced accessibility of P-glycoprotein to the dexniguldipine action (and not competition for the binding site), probably because of an increased membrane fluidity under the influence of ilmofosine. The data also demonstrate that the MDR-modulating potency of dexniguldipine-HCl is not reduced in the presence of a tenfold molar excess of the phospholipid analogue. On the contrary, there is a potentiation of the inhibitory effect of dexniguldipine-HCl.

That ilmofosine indeed enhances the accessibility of P-glycoprotein ligands is also evident from photoaffinity studies. The dihydropyridine azidopine is a well-established photoaffinity label for P-glycoprotein. Figure 4 demonstrates that there is no competition between ilmofosine and [$^3$H]azidopine. On the contrary, ilmofosine in this experiment also renders P-glycoprotein more accessable and increases the binding of azidopine. Furthermore, no significant alteration of the P-glycoprotein ATPase activity by ilmofosine could be detected, also indicating no direct interaction of ilmofosine with P-glycoprotein (Figure 5).

### Resistance of Meth A cells

As described above, multidrug-resistant cells are cross-resistant to ilmofosine. The question was whether cells selected for resistance to ilmofosine are multidrug resistant and therefore cross-resistant to drugs transported by P-glycoprotein. In order to investigate this question, we used murine Meth A fibrosarcoma cells sensitive and resistant to ilmofosine. The resistance was obtained by addition of increasing concentrations of the phospholipid analogue ET-18-$OCH_3$ (1-octadecyl-2-*O-rac*-glyero-3-phosphocholine) (Petersen et al, 1992). The cells are cross-resistant to ilmofosine and have been grown in the presence of 6 μg ml$^{-1}$ (equivalent to 11.7 μM) ilmofosine for long periods of time. Dose–response curves of the sensitive and resistant cell lines are shown in Figure 6. Both cell lines exhibit similar sensitivity to vinblastine (Figure 7), indicating that long-term treatment with ilmofosine does not lead to a multidrug-resistant phenotype. Also short-term treatment with the compound does not increase the *MDR1* mRNA levels, as shown in Figure 8.

### Expression of the MDR2 gene

It has been reported recently that the *MDR2*-encoded P-glycoprotein transports phosphatidylcholine out of the cell (Smit et al, 1993; Ruetz et al, 1994). For this reason, we investigated whether this gene is involved in the resistance to ilmofosine by detection of the *MDR1* and *MDR2* mRNA levels. In Figure 9, the amount of *MDR1* mRNA and that of the *MDR2* mRNA in the different cell lines is shown. In sensitive HeLa-WT and resistant *MDR1*-overexpressing HeLa cells, no *MDR2* expression was observed. Both MCF-7 cell lines exhibit similar levels of *MDR2* mRNA (Figure 9). These results argue against a role of *MDR2* in the resistance to ilmofosine.

### DISCUSSION

Our results show that the *MDR1*-overexpressing cells MCF-7/ADR, CEM/VLB$_{100}$, CCRF/VCR1000, CCRF/ADR5000 and two HeLa cell lines transfected with *MDR1* genes are cross-resistant to ilmofosine. Although the resistance to ilmofosine is considerably lower than to vinblastine or doxorubicin, a three- to sevenfold resistance can cause treatment failure in patients. Altered toposiomerase II-resistant CEM/VM-1 cells do not exhibit cross-resistance to the compound. The data obtained with HeLa-MDR1-G185 and HeLa-MDR1-V185 cells indicate that elevation of the expression of *MDR1* by transfection of the gene is sufficient to elicit cross-resistance to ilmofosine and that no additional mechanism seems to be involved. P-glycoprotein transports lipophilic compounds out of the cell. Therefore, cross-resistance of multidrug-resistant cells could be caused by increased efflux of ilmofosine catalysed by the P-glycoprotein. Our data argue against this possibility: (1) ilmofosine does not enhance the antiproliferative activity of doxorubicin (Figure 1A and B) or vinblastine (data not shown); (2) the potent P-glycoprotein modulator dexniguldipine-HCl is not able to affect the sensitivity to ilmofosine (Figure 2); (3) a tenfold excess of ilmofosine does not compete with the P-glycoprotein inhibitory effect of dexniguldipine-HCl, as shown by the intracellular accumulation of rhodamine 123 (Figure 3); (4) ilmofosine does not compete with azidopine for the binding site (Figure 4); and (5) the ATPase activity determined in presence of ilmofosine is not altered significantly (Figure 5). This is an indication that ilmofosine does not interact directly with the P-glycoprotein, a conclusion that has recently also been reported by Principe et al (1994). A possible explanation for cross-resistance of multidrug-resistant cells to ilmofosine is that P-glycoprotein-expressing cells have an altered lipid composition, which renders cells less susceptible to the antiproliferative activity of ilmofosine (Endicott and Ling, 1989). In addition, P-glycoprotein seems to be involved in the transport of sterols from the membrane to the endoplasmic reticulum (Metherall et al, 1996); this may lead to altered lipid composition of membranes, leading to resistance to ilmofosine. It was reported previously that a unique glycosphingolipid pattern is associated with multidrug resistance in MCF7 cells (Lavie et al, 1996). Le Moyec et al (1996) showed by nuclear magnetic resonance spectroscopy that cellular lipids are involved in the *MDR1*-mediated resistance to doxorubicin and taxol. Our results are consistent with these reports. Resistance of *MDR1*-expressing cells to ilmofosine seems not to be a direct but an indirect effect of P-glycoprotein action. *MDR1* expression leads to lipid and membrane alterations, and these alterations seem to cause resistance to ilmofosine.

It was shown that phosphatidylcholine is transported by the *MDR2*-encoded P-glycoprotein (Smit et al, 1993; Ruetz et al, 1994). Because ilmofosine is a phosphatidylcholine analogue, it could be speculated that *MDR2* is responsible for differences in the sensitivity to the compound. Our results argue against this possibility (Figure 9). Thorgeirsson et al (1991) proposed a possible mechanism for co-induction of the *MDR1* and *MDR2* gene. We could not observe a co-expression of both genes in our cell lines (Figure 9).

Ilmofosine is an inhibitor of protein kinase C (Hofmann et al, 1989). There is experimental evidence that phosphorylation of P-glycoprotein by protein kinase C modulates the activity of the efflux pump (Ma et al, 1991; Chambers et al, 1992; Ahmad and Glazer, 1993). For the protein kinase C inhibitor ilmofosine, this mechanism seems to be of minor importance because the compound does not reduce multidrug resistance (Figure 1A and B; data not shown). KB-8-5 cells are the only cells in which ilmofosine is able to reverse multidrug resistance. These cells are also not cross-resistant to ilmofosine (data not shown). The reason may be that KB cells are exceptionally sensitive to phospholipid analogues (Fleer et al, 1993).

It has been reported that modulation of protein kinase C activity may alter the expression of the *MDR1* gene (Chaudhary and Roninson, 1992; Sampson et al, 1993). Ilmofosine, however, does not influence the expression of the *MDR1* gene as detected by polymerase chain reaction (Figure 8). Long-term exposure of cells to ilmofosine, as shown in MR cells that are grown in the presence of the drug, did not lead to a multidrug-resistant phenotype. As shown in Figure 7, there is no difference in sensitivity to vinblastine in MS and MR cells. From these experiments, it can be concluded that treatment of patients with ilmofosine may not induce multidrug resistance. Multidrug resistance in patients is usually in the range of MCF7/ADR cells or lower. As shown in Table 1, the resistance of the MCF7/ADR cell line to ilmofosine is in the same range as doxorubicin. Therefore, the question whether expression of the MDR phenotype in patients may influence the effects of ilmofosine treatment in the clinic has to be addressed.

### ACKNOWLEDGEMENTS

This work was supported by the grant P10664-MED from the Austrian Science Foundation. WT Beck is supported in part by research grants CA 40570 and CA3010, programme grant CA23099 and cancer center support (CORE) grant CA21765, all from the National Cancer Institute, Bethesda, MD, USA, and in part by American Lebanese Syrian Associated Charities.

### ABBREVIATIONS

Dexniguldipine-HCl, B8509-035, (4R)-3-[4,4-diphenyl-1-piperidinyl(propyl)]-5-methyl-1,4-dihydro-2,6-dimethyl-4-(3-nitrophenyl)-pyridine-3,5-dicarboxylate-hydrochloride; EGTA, ethyleneglycol-bis(β-aminoethylether) *N,N,N′,N′*-tetraacetic acid); HeLa-WT, drug-sensitive HeLa wild-type cells; HeLa-MDR1-G185, HeLa-WT cells transfected with a *MDR1* wild-type gene containing a glycine in position 185; HeLa-MDR1-V185, HeLa-WT cells transfected with a mutant *MDR1* gene containing a valine in position 185; ilmofosine, BM 41 440, 3-hexadecyl-mercapto-2-methoxymethyl-propyl-1-phosphocholine; MDR, multidrug resistance; *MDR1*, multidrug resistance gene 1; *MDR2*, multidrug resistance gene 2; MS, Meth A cells sensitive to ilmofosine; MR, Meth A cells resistant to ilmofosine; PCR, polymerase chain reaction

### REFERENCES

Ahmad S and Glazer RI (1993) Expression of the antisense cDNA for protein kinase Cα attenuates resistance in doxorubicin-resistant MCF-7 breast carcinoma cells. *Mol Pharmacol* **43**: 858–862

Beck WT, Mueller TJ and Tanzer LR (1979) Altered surface membrane glycoproteins in *Vinca* alkaloid-resistant human leukemic lymphoblasts. *Cancer Res* **39**: 2070–2076

Berdel WE (1991) Membrane-interactive lipids as experimental anticancer drugs. *Br J Cancer* **64**: 208–211

Chambers TC, Zheng B and Kuo JF (1992) Regulation by phorbol ester and protein kinase C inhibitors, and by a protein phosphatase inhibitor (okadaic acid), of P-glycoprotein phosphorylation and relationship to drug accumulation in multidrug-resistant human KB cells. *Mol Pharmacol* **41**: 1008–1015

Chaudhary PM and Roninson IB (1992) Activation of MDR1 (P-glycoprotein) gene expression in human cells by protein kinase C agonists. *Oncol Res* **4**: 281–290

Danks MK, Schmidt CA, Cirtain MC, Shuttle DP and Beck WT (1988) Altered catalytic activity of and DNA cleavage by topoisomerase II from human leukemic cells selected for resistance to VM-26. *Biochemistry* **27**: 8861–8869

Doige CA, Yu X and Sharom FF (1992) ATPase activity of partially purified P-glycoprotein from multidrug-resistant Chinese hamster ovary cells. *Biochim Biophys Acta* **1109**: 149–160

Endicott JA and Ling V (1989) The biochemistry of P-glycoprotein-mediated multidrug resistance. *Annu Rev Biochem* **58**: 137–171

Fleer EAM, Berkovic D, Eibl H and Unger C (1993) Investigations on the cellular uptake of hexadecylphosphocholine. *Lipids* **28**: 731–736

Gottesman MM and Pastan I (1993) Biochemistry of multidrug resistance by the multidrug transporter. *Annu Rev Biochem* **62**: 385–427

Hamada H and Tsuruo T (1988) Purification of 170–180 kilodalton membrane P-glycoprotein associated with multidrug resistance. *J Biol Chem* **263**: 1454–1458

Herrmann DBJ (1985) Changes in cellular lipid synthesis of normal and neoplastic cells during cytolysis induced by alkyl lysophospholipid analogues. *J Natl Cancer Inst* **75**: 423–430

Himmelmann AW, Dannhauser-Riedl S, Steinhauser G, Busch R, Modest EJ, Noseda A, Rastetter J, Vogler RW and Berdel WE (1990) Cross-resistance pattern of cell lines selected for resistance towards different cytotoxic drugs to membrane-toxic phospholipids *in vitro*. *Cancer Chemother Pharmacol* **26**: 437–443

Hofmann J, Ueberall F, Posch L, Maly K, Hermann DBJ and Grunicke H (1989) Synergistic enhancement of the antiproliferative activity of cis-diamminedichloro-platinum(II) by the ether lipid analogue BM41440, an inhibitor of protein kinase C. *Lipids* **24**: 312–317

© Cancer Research Campaign 1997

Hofmann J, Wolf A, Spitaler M, Böck G, Drach J, Ludescher C and Grunicke H (1992) Reversal of multidrug resistance by B859-35, a metabolite of B859-35, niguldipine, verapamil and nitrendipine. *J Cancer Res Clin Oncol* **118**: 381–366

Hofmann J, O'Connor PM, Jackman J, Schubert C, Ueberall F, Kohn KW and Grunicke H (1994) The protein kinase C inhibitor ilmofosine (BM 41 440) arrests cells in G2 phase and suppresses CDC2 kinase activation through a mechanism different from that of DNA damaging agents. *Biochem Biophys Res Commun* **199**: 937–943

Kane SE, Reinhard DH, Fordis MC, Pastan I and Gottesman MM (1989) A new vector using the human multidrug resistance gene as a selectible marker enables overexpression of foreign genes into eukaryotic cells. *Gene* **84**: 439–446

Kimmig A, Gekeler V, Neumann M, Frese G, Handgretinger R, Kardos G, Diddens H and Niethammer D (1990) Susceptibility of multidrug-resistant human leukemia cell lines to human interleukin 2-activated killer cells. *Cancer Res* **50**: 6793–6799

Lavie Y, Ca H, Bursten SL, Armando GE and Cabot MC (1996) Accumulation of glucosylceramides in multidrug-resistant cancer cells. *J Biol Chem* **271**: 19530–19536

Le Moyec L, Tatoud R, Degeorges A, Calabresse C, Bauza G, Euene M and Calvo F (1996) Proton nuclear magnetic resonance spectroscopy reveals cellular lipids involved in resistance to adramycin and taxol by the K562 leukemia cell line. *Cancer Res* **56**: 3461–3467

Ma L, Marquart D, Takemoto L and Center MS (1991) Analysis of P-glycoprotein phosphorylation in HL60 cells isolated for resistance to vincristine. *J Biol Chem* **266**: 5593–5599

Metherall JE, Li H and Waugh K (1996) Role of multidrug resistance P-glycoproteins in cholesterol biosynthesis. *J Biol Chem* **271**: 2634–2640

Noonan KE, Beck C, Holzmayer TA, Chin JE, Wunder JS, Andrulis IL, Gazdar AF, Willman CL, Griffith B, Von Hoff DD and Roninson IB (1990) Quantitative analysis of MDR1 (multidrug resistance) gene expression in human tumors by polymerase chain reaction. *Proc Natl Acad Sci USA* **87**: 7160–7164

Petersen ES, Kelley EE, Modest EJ and Burns PC (1992) Membrane lipid modification and sensitivity of leukemic cells to the thioether lipid analogue BM 41.440. *Cancer Res* **52**: 6263–6269

Powis G, Seewald MJ, Gratas C, Melder D, Riebow J and Modest EJ (1992) Selective inhibition of phosphatidylinositol phospholipase-C by cytotoxic ether lipid analogs. *Cancer Res* **52**: 2835–2840

Principe P, Coulomb H, Broquet C and Braquet P (1992) Evaluation of combinations of antineoplastic ether lipids and chemotherapeutic drugs. *Anti-Cancer Drugs* **3**: 577–587

Principe P, Fausat-Suberville AM, Coulomb H, Marie J-P and Braquet P (1994) Flow cytometric monitoring of anthracycline accumulation after anti-neoplastic ether phospholipid treatment. *Anti-Cancer Drugs* **5**: 329–335

Ruetz S and Gros P (1994) Phosphatidylcholine translocase: a physiological role for the mdr2 gene. *Cell* **77**: 1071–1081

Sampson E, Wolff C and Abraham I (1993) Staurosporine reduces P-glycoprotein expression and modulates multidrug resistance. *Cancer Lett* **68**: 7–14

Skehan P, Storeng R, Scudiero D, Monks A, McMahon J, Vistica D, Warren JT, Bokesch H, Kenney S and Boyd MR (1990) New colorimetric cytotoxicity assay for anticancer-drug screening. *J Natl Cancer Inst* **82**: 1107–1112

Smit JJM, Schinkel AH, Oude Elferink RPJ, Groen AK, Wagenaar E, Van Deemter L, Mol Caam, Ottenhoff R, Van Der Lugt NMT, Van Roon MA, Van Der Valk MA, Offerhaus GJA, Berns AJM and Porst P (1993) Homozygous disruption of the murine mdr2 P-glycoprotein gene leads to a complete absence of phospholipid from bile to liver disease. *Cell* **75**: 451–462

Thorgeirsson SS, Silverman JA, Gant TW and Marino PA (1991) Multidrug resistance gene family and chemical carcinogenesis. *Pharmaceut Ther* **49**: 283–292.

Winkelmann M, Ebeling K, Strohmeyer G, Hottenrott G, Mechl Z, Berges W, Scholten T, Westerhausen M, Schlimok G and Sterz R (1992) Treatment results of the thioether lipid ilmofosine in patients with malignant tumours. *J Cancer Res Clin Oncol* **118**: 405–407

© Cancer Research Campaign 1997